# Effect of exercise prescription intervention mode in the Omaha System in elderly patients with delayed gastric emptying after choledocholithiasis surgery

**DOI:** 10.3389/fsurg.2025.1667853

**Published:** 2025-10-30

**Authors:** Wenjing Kan, Huacui Sun, Ruimei Chen

**Affiliations:** First Department of General Surgery, Suzhou Hospital of Anhui Medical University, Suzhou, Anhui, China

**Keywords:** delayed gastric emptying, choledocholithiasis surgery, elderly, Omaha System, exercise prescription

## Abstract

**Objective:**

This study aimed to explore the impact of exercise prescription intervention mode according to the Omaha System on defecation recovery in elderly patients with delayed gastric emptying (DGE) after choledocholithiasis surgery.

**Methods:**

A total of 96 elderly patients with DGE after choledocholithiasis surgery admitted to our hospital from July 2019 to June 2022 were selected and split into the control group (CG) and observation group (OG). The CG adopted a routine nursing intervention. Based on the CG, patients in the OG adopted an exercise prescription intervention based on the Omaha System. The postoperative defecation recovery time, negative emotions, sleep quality, quality of life, and nursing satisfaction of patients in both groups were compared.

**Results:**

Relative to the CG, the postoperative defecation recovery time of the OG was shorter (*P* < 0.05). Self-rating anxiety scale, self-rating depression scale, and Pittsburgh sleep quality index scores in the OG were lower compared with the CG after intervention (*P* < 0.05). Each dimension of the 36-item short form score in the OG was higher compared with the CG after intervention (*P* < 0.05). The nursing satisfaction of patients in the OG was higher compared with the CG (*P* < 0.05).

**Conclusion:**

The Omaha System-based exercise prescription intervention significantly accelerated gastrointestinal function recovery and improved quality of life in elderly patients with postoperative DGE, suggesting it is a valuable and recommended adjunct to routine postoperative care.

## Introduction

Choledocholithiasis is a common clinical disease of the biliary tract, with an incidence of approximately 8%–10% in China (1, 2). With the continuous improvement of people's life quality and the increase in life and work pressure, the incidence of cholelithiasis shows an increasing trend year by year (3), and the incidence of elderly choledocholithiasis also shows an increasing trend, with the incidence of people over 70 years old reaching 48% (4–6). At present, laparoscopic surgery has been widely adopted in choledocholithiasis because of its advantages of less trauma, quick recovery, and short hospital stay (7). Owing to the progressive decline in physiological reserve across multiple organ systems, elderly patients exhibit reduced surgical tolerance and are more susceptible to postoperative complications such as delayed gastric emptying (DGE) (8). Research results have shown that postoperative planned exercise training for patients is conducive to gastrointestinal function recovery (9, 10).

Exercise prescription is a diagnostic prescription of exercise items, exercise intensity, exercise time, and frequency suitable for individuals based on the study of individual health and physical functions and the characteristics of exercise items (11, 12). The exercise prescription provides detailed, evidence-based guidance for postoperative rehabilitation, enabling nurses to deliver standardized care and empowering patients to clearly understand and adhere to their daily exercise regimen, standardizing postoperative rehabilitation guidance, and accelerating the recovery of diseases (13, 14). Studies have revealed that exercise prescription has a certain promoting role in physical health, which can improve some diseases and restore health (15). It has been widely used in cardiovascular system, respiratory disease, and nervous system disease (5, 16–18). However, it is rarely used in elderly patients with delayed gastric emptying after choledocholithiasis surgery.

The Omaha System is a simplified nursing procedure mode through the use of health education, operation procedures, case management, and supervision and evaluation processes to intervene and manage patients (19). It has significant clinical effects on improving patients' cognition, behavior, status, and quality of life (20). However, the application of the Omaha System in elderly patients with delayed gastric emptying after choledocholithiasis surgery is rare.

Despite the established efficacy of exercise prescription and the Omaha System in their respective fields, the integration of both into a structured intervention model, specifically tailored for elderly patients with DGE following choledocholithiasis surgery, remains scarcely explored. Previous research on postabdominal surgery rehabilitation has largely focused on colorectal or gastrectomy surgeries or adhered to the general principles of Enhanced Recovery After Surgery (ERAS) protocols, lacking highly specific exercise interventions targeting a particular complication of biliary tract surgery—namely, DGE. The novelty of the present study lies in its pioneering integration of the Omaha System's problem classification and intervention framework with individualized exercise prescription to design and validate a systematic, operable nursing intervention model for this specific patient population. This approach not only provides a concrete exercise regimen but also, through the Omaha System, ensures the comprehensiveness and individualization of the intervention. It is therefore posited that this study offers a novel, theoretically grounded methodology to address this clinical challenge.

Hence, our study aimed to probe the impact of exercise prescription intervention according to the Omaha System on defecation recovery in elderly patients with delayed gastric emptying after choledocholithiasis surgery.

## Data and methods

### Study design

This study was a prospective, randomized controlled trial conducted at the First Department of General Surgery, Suzhou Hospital of Anhui Medical University. A total of 96 elderly patients with DGE after choledocholithiasis surgery who were hospitalized in our hospital from July 2019 to June 2022 were enrolled as the research subjects using a convenience sampling method. Patients were randomly assigned to either the control group (CG) or the observation group (OG), with 48 cases in each group. The CG contained 26 males and 22 females, aged from 60 to 80 years old, and the mean age was (69.32 ± 7.28) years old. The diameter of the stone was 6–23 mm, with an average of (14.42 ± 2.35) mm. The OG contained 27 males and 21 females, aged from 61 to 82 years old, and the mean age was (69.35 ± 7.31) years old. The diameter of the stone was 6–24 mm, with an average of (14.48 ± 2.37) mm. The above general data were of no significance in both groups, indicating comparable (*P* > 0.05; Table 1).

**Table 1:** Comparison of baseline characteristics between control and observation groups.

Characteristic	Control group (*n* = 48)	Observation group (*n* = 48)	*P*
Age (years)			
Mean ± SD	69.32 ± 7.28	69.35 ± 7.31	0.984
Gender, *n* (%)			
Male	26 (54.2%)	27 (56.3%)	0.840
Female	22 (45.8%)	21 (43.7%)	
Stone diameter (mm)			
Mean ± SD	14.42 ± 2.35	14.48 ± 2.37	0.899
Comorbidities, *n* (%)			
Hypertension	18 (37.5%)	16 (33.3%)	0.680
Diabetes mellitus	9 (18.8%)	11 (22.9%)	0.620
Operation time (min)			
Mean ± SD	125.6 ± 25.3	121.8 ± 23.7	0.432
Preoperative labs			
Hemoglobin (g/L)	132.5 ± 14.2	130.8 ± 15.6	0.557
Albumin (g/L)	38.5 ± 3.2	39.1 ± 3.5	0.371

Continuous variables were compared using an independent samples *t*-test; categorical variables were compared using a *χ*^2^ test.

### Participants

Inclusion criteria: (1) patients aged ≥60 years; (2) diagnosed with choledocholithiasis by ultrasonography or magnetic resonance cholangiopancreatography before surgery; (3) with delayed gastric emptying after choledocholithiasis surgery; (4) have no neurological, muscular, joint, or other diseases affecting movement; and (5) have informed consent and willingness to take part in this study. Exclusion criteria: (1) patients with severe heart, liver, and kidney disease; (2) mental illness or consciousness disorder; and (3) poor compliance.

### Randomization, allocation concealment, and blinding

Participants were randomly allocated in a 1:1 ratio to the CG or OG using a computer-generated random number sequence by an independent statistician who was not involved in participant recruitment or intervention. The allocation sequence was concealed by placing it in sequentially numbered, opaque, sealed envelopes (SNOSE) to ensure allocation concealment. The envelopes were opened by a research nurse only after the enrolled participant had completed all baseline assessments.

Due to the nature of the behavioral intervention, it was not feasible to blind the patients or the nursing staff administering the care. However, to minimize assessment bias, the research assistants who collected the outcome data [including administering the self-rating anxiety scale (SAS), self-rating depression scale (SDS), Pittsburgh sleep quality index (PSQI), and 36-item short form (SF-36) questionnaires] were blinded to the group assignment throughout the study period.

### Sample size calculation

The sample size was determined *a priori* using G*Power software (version 3.1.9.7). Based on preliminary data from our institution, the primary outcome was the time to recovery of defecation. To detect an anticipated mean difference of 12 h in defecation time between groups with a standard deviation (SD) of 18 h, an effect size (*d*) of 0.67 was calculated. With a significance level (*α*) of 0.05 and a desired power (1 − *β*) of 0.80 for a two-tailed independent *t*-test, the analysis indicated a required sample size of 36 participants per group. To account for a potential dropout rate of approximately 15%, we aimed to recruit 48 participants per group, yielding a total sample size of 96.

### Interventions

Patients in the CG adopted routine nursing intervention. The routine nursing included monitoring of vital signs, dietary guidance, medication management, and encouragement to get out of bed as soon as possible (but without structured, supervised exercise programs).

On the basis of the CG, patients in the OG adopted an exercise prescription intervention based on the Omaha System. The specific contents were as follows. (1) An exercise prescription intervention group was established based on the Omaha System. The group was composed of 10 nurses, 1 chief nurse, and 1 doctor with more than 5 years' working experience and nursing titles or above. Before the implementation of exercise prescription intervention, members of the group were trained on the concept, use methods, and evaluation tools of the Omaha System and theoretical knowledge of treatment and nursing of choledocholithiasis, to ensure that each member of the group could master the procedure of using the Omaha System and its significance. (2) Specific implementation of exercise prescription intervention based on the Omaha System:

a) Team members should understand and determine the treatment situation and psychological and physiological changes of patients; analyze the problems of patients from the physiological, health, psychological, social, and other fields; classify the problems into 42 items through the Omaha problem classification system; and formulate the corresponding rehabilitation treatment plan based on the specific health problems of patients.

b) Exercise prescription intervention: The patient was instructed to get out of bed and move in the order of sitting up on the bed, standing beside the bed, and walking on the ground. The principle of activities was gradual. After a circle of activity around the bed, the patient could take a rest for a while, once in the morning, once in the afternoon, and once in the evening, 15–20 min each time.

### Outcome measures

1. Postoperative defecation recovery time was recorded in both groups.
2. The negative emotions of both groups were assessed by virtue of the SAS and SDS (21).
3. The sleep quality of patients in both groups was assessed based on the PSQI (22, 23).
4. The quality of life of patients was assessed by SF-36 (24, 25), which mainly included patients’ physiological function, mental health, role physical, body pain, energy, emotional function, social function, and general health.
5. Nursing satisfaction: the use of a self-compiled nursing satisfaction questionnaire evaluation. The evaluation results were divided into unsatisfied, basically satisfied, satisfied, and very satisfied. The final evaluation of the overall satisfaction of nursing: the cumulative evaluation score of each item satisfaction/total cases × 100%.

### Statistical analysis

All statistical analyses were performed using SPSS Statistics version 20.0 (IBM Corp., Armonk, NY, USA). The normality of the distribution for continuous variables was assessed using the Shapiro–Wilk test, and the homogeneity of variances was verified using Levene's test. Data were presented according to their distribution and type. Continuous variables were expressed as mean ± SD. Categorical variables were expressed as numbers and percentages (*n*, %). Between-group comparisons at baseline for demographic and clinical characteristics were conducted using independent samples *t*-tests for normally distributed continuous variables and Chi-square (*χ*^2^) tests or Fisher's exact test for categorical variables, as appropriate.

For the analysis of outcome measures, the primary outcome, postoperative defecation recovery time, was compared between the CG and OG using an independent samples *t*-test. The mean difference between groups along with its 95% confidence interval (95% CI) was reported. For secondary outcomes measured at both baseline and post-intervention (SAS, SDS, PSQI, and all SF-36 domains), comparisons were performed using a two-way repeated-measures analysis of variance (ANOVA). This model included one between-subjects factor (group: CG vs. OG) and one within-subjects factor (time: pre- vs. post-intervention). The primary focus was the significant group–time interaction effect, which indicates that the change over time differed between the two groups. If a significant interaction was found, simple effects analyses (independent and paired *t*-tests with Bonferroni correction) were conducted to pinpoint the differences. Nursing satisfaction (ordinal categorical data) was compared between groups using the Mann–Whitney *U* test.

Adjustment for multiple comparisons: Given the multiple comparisons involved in the analysis of the eight SF-36 domains, a Bonferroni correction was applied to control for the family-wise error rate. The significance level for these eight comparisons was therefore adjusted to (*P* < 0.00625).

Reporting of *P*-values and CIs: Exact *P*-values are reported throughout, and *P* < 0.05 was considered statistically significant, except where adjusted for multiple comparisons. For key between-group comparisons, particularly for the primary outcome, 95% CIs are provided to give an estimate of the precision and clinical relevance of the observed effects.

## Results

### Primary outcome: postoperative defecation recovery time in both groups

**Figure 1:**
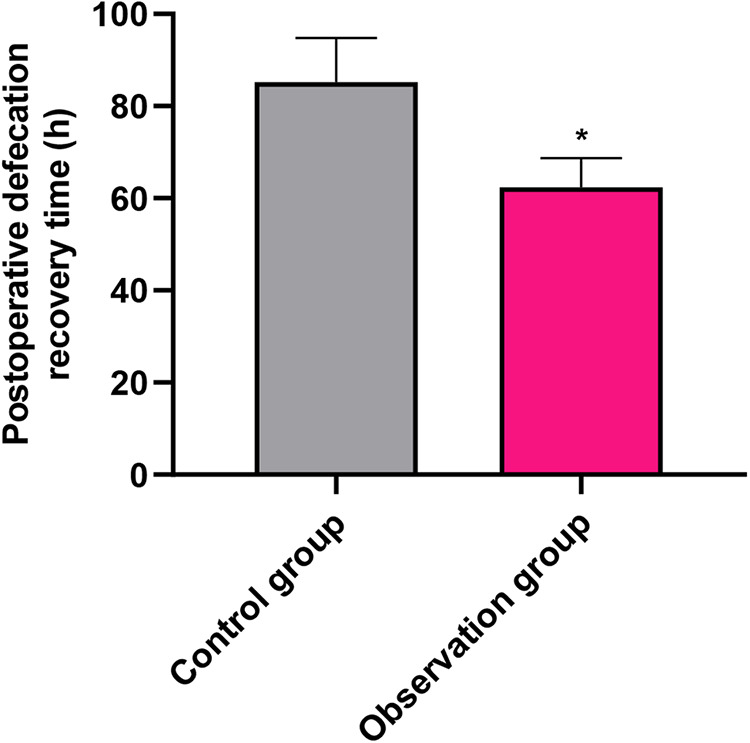
Comparison of postoperative defecation recovery time (primary outcome) between the control group (CG) and observation group (OG). Data are presented as mean ± standard deviation. *P < 0.001.

The time to recovery of defecation, the primary outcome of this study, was significantly shorter in the OG compared with the CG (62.37 ± 6.31 h vs. 85.23 ± 9.52 h; mean difference, −22.86 h; 95% CI, −26.14 to −19.58; *P* < 0.001; Figure 1). This represents a clinically meaningful acceleration in gastrointestinal function recovery, reducing the recovery time by approximately 27%.

### Secondary outcomes: psychological status and sleep quality

**Figure 2:**
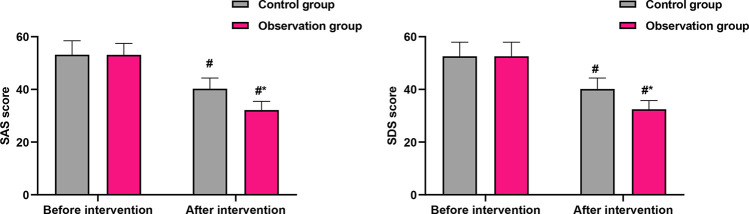
Comparison of SAS and SDS scores before and after intervention. Data are presented as mean ± standard deviation. ^#^*P* < 0.001, compared with before intervention within the same group; *P < 0.001, compared with the control group at the same time point.

**Figure 3:**
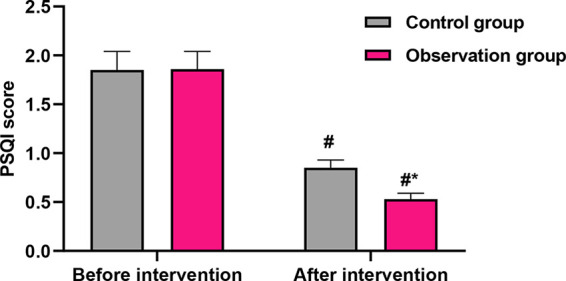
Comparison of PSQI scores before and after intervention. Data are presented as mean ± standard deviation. ^#^*P* < 0.001, compared with before intervention within the same group; *P < 0.001, compared with the control group at the same time point.

No significant differences were found in SAS, SDS, or PSQI scores between the two groups at baseline (*P* > 0.05), indicating comparability before the intervention. Following the intervention, both groups showed improvements; however, the OG demonstrated statistically superior outcomes. Anxiety (SAS): The post-intervention SAS score in the OG was significantly lower than that in the CG (32.17 ± 3.28 vs. 40.26 ± 4.08, *P* < 0.001; Figure 2). This difference of 8.09 points exceeds the commonly accepted minimum clinically important difference for the SAS, indicating a meaningful reduction in anxiety symptoms among intervention recipients. Depression (SDS): Similarly, the post-intervention SDS score was significantly lower in the OG compared with the CG (32.47 ± 3.28 vs. 40.18 ± 4.15, *P* < 0.001; Figure 2). The 7.71-point between-group difference suggests a substantial alleviation of depressive symptoms in the OG. Sleep quality (PSQI): The PSQI score improved significantly more in the OG after the intervention (0.53 ± 0.06 vs. 0.85 ± 0.08, *P* < 0.001; Figure 3), indicating better sleep quality. The final PSQI score in the OG (0.53) is well below the clinical threshold for poor sleep quality, underscoring the intervention's strong effect on normalizing sleep patterns.

### Secondary outcomes: health-related quality of life (SF-36)

**Figure 4:**
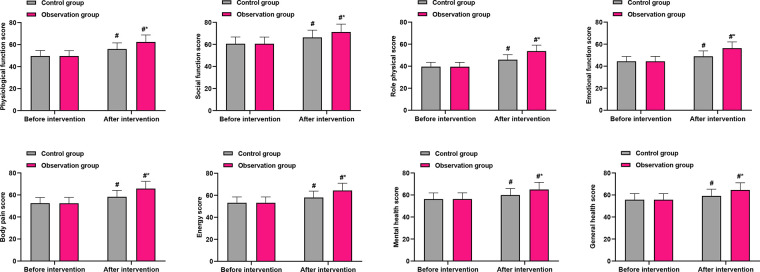
Comparison of SF-36 quality of life domain scores between groups after intervention. PF, physical functioning; RP, role-physical; BP, bodily pain; GH, general health; VT, vitality; SF, social functioning; RE, role-emotional; MH, mental health. Data are presented as mean ± standard deviation. *P < 0.001 for all between-group comparisons (OG vs. CG) post-intervention.

Baseline scores across all eight domains of the SF-36 were comparable between groups (*P* > 0.05). After the intervention, patients in the OG showed significantly greater improvement in all domains compared with the CG (*P* < 0.001 for all domains; Figure 4). The magnitude of this between-group benefit is visually summarized in Figure 4. The most substantial between-group differences (exceeding six points) were observed in physical function (OG: 62.38 ± 6.35 vs. CG: 55.89 ± 5.62), role-physical (OG: 53.67 ± 5.41 vs. CG: 45.87 ± 4.62), bodily pain (OG: 65.78 ± 6.59 vs. CG: 58.32 ± 5.84), and general health (OG: 64.58 ± 6.51 vs. CG: 59.18 ± 6.03). These domains, which reflect core aspects of physical health, appear to have derived the greatest benefit from the exercise prescription intervention.

### Nursing satisfaction of patients in both groups

As detailed in Table 2, the overall satisfaction rate was 95.83% in the OG, markedly higher than the 79.17% observed in the CG. This 16.66% absolute increase underscores the high acceptability of the Omaha System-based intervention from the patients' perspective.

**Table 2:** Comparison of nursing satisfaction of patients between control and observation groups.

Groups	Very satisfied	Satisfied	Basically satisfied	Unsatisfied	Total incidence rate
Control group (*n* = 48)	20	8	10	10	38 (79.17%)
Observation group (*n* = 48)	30	10	6	2	46 (95.83%)
*χ* ^2^	6.10				
*P*	<0.05				

## Discussion

The principal and most clinically significant finding of this randomized controlled trial is that the Omaha System-based exercise prescription intervention significantly shortened the time to recovery of defecation—the primary outcome—in elderly patients suffering from DGE after choledocholithiasis surgery. The observed reduction of nearly 23 h (approximately 27%) is not only statistically robust but also holds substantial clinical importance, as it directly addresses a common and debilitating postoperative complication that prolongs hospital stay and increases patient discomfort (26, 27).

Our findings provide strong empirical support for integrating a structured, theory-driven exercise intervention within the postoperative care protocol for this specific patient population. They effectively operationalize the core ERAS principle of early and structured mobilization (28). While ERAS protocols have been widely adopted, their components' efficacy can vary across different surgical populations. Our study contributes novel evidence by demonstrating that a systematized exercise prescription, embedded within a comprehensive care framework like the Omaha System, is particularly effective in mitigating DGE in elderly biliary surgery patients—a group highly vulnerable to postoperative complications due to age-related decline in physiological reserve and gastrointestinal motility (29). This approach moves beyond generic “early mobilization” advice to a personalized, problem-based strategy, aligning with the growing emphasis on tailored rehabilitative interventions in geriatric surgery (30).

The pronounced effect on gastrointestinal recovery can be attributed to several interconnected physiological mechanisms, which our intervention likely engaged. Neurohormonal modulation: Physical activity, even of mild to moderate intensity, is known to enhance vagal tone and stimulate the release of gastrointestinal hormones such as motilin, which are critical for coordinating gastroduodenal motility and the migrating motor complex. The structured walking regimen may have served as a potent physiological stimulus to restart these stalled patterns of motility, directly countering the pathophysiology of DGE. Mechanical stimulation: The act of assuming an upright posture and ambulating provides gravitational and mechanical stimulation to the abdominal cavity. This can promote the propulsive activity of the intestines and help prevent the pooling of secretions and gas, thereby facilitating the aboral movement of content and the return of peristalsis. Systemic and metabolic effects: Exercise improves systemic blood flow, including splanchnic perfusion, which is crucial for healing and functional recovery of the gastrointestinal tract. Furthermore, by mitigating the surgical stress response and reducing systemic inflammation, exercise may create a more favorable metabolic environment for organ function restoration (31).

Beyond the primary gastrointestinal outcome, our study revealed meaningful benefits in several patient-centered secondary outcomes. Patients receiving the Omaha System-based exercise intervention reported significantly greater reductions in anxiety and depression scores, alongside markedly improved sleep quality and quality of life. We hypothesize that these benefits were mediated indirectly through several pathways: the psychological reassurance and sense of agency fostered by a clear, structured recovery plan; the inherent anxiolytic and mood-stabilizing neurobiological effects of regular physical activity (32); and the subsequent alleviation of physical distress and worry as core gastrointestinal symptoms resolved. This interconnected improvement likely contributed to the observed, superior gains in overall health-related quality of life, creating a positive feedback loop that enhanced both physical and psychological convalescence. This underscores the value of the Omaha System in addressing the patient's recovery experience comprehensively, beyond a sole focus on physical symptoms.

Several limitations of this study should be acknowledged. First, the single-center design may limit the generalizability of our findings, and replication in multicenter trials is warranted. Second, while outcome assessors were blinded, the nature of the behavioral intervention prevented the blinding of patients and care providers, which could introduce potential performance bias. Future research should also explore the long-term sustainability of these benefits. Third, the use of a convenience sampling method from a single center may limit the generalizability of our findings to broader populations or different clinical settings. Although our sample size was determined by an *a priori* power calculation and was sufficient for the primary outcome, future multicenter studies with larger, consecutive samples are warranted to confirm and extend our results.

## Conclusion

Exercise prescription intervention based on the Omaha System could promote recovery of gastrointestinal function and improve the quality of life of elderly patients with DGE after choledocholithiasis surgery, which is worthy of promotion.

## Data Availability

The original contributions presented in the study are included in the article/Supplementary Material, further inquiries can be directed to the corresponding author.
